# Late corneal edema due to retained foldable lens fragment

**DOI:** 10.4103/0301-4738.49401

**Published:** 2009

**Authors:** Nikhil S Gokhale

**Affiliations:** Gokhale Eye Hospital and Eyebank, Anant Building, Gokhale Road (S), Dadar West, Mumbai-400 028, India

**Keywords:** Corneal edema, fracture, intraocular lens

## Abstract

Late onset of corneal edema after cataract surgery is an unusual complication. We report a case of corneal edema presenting one month after cataract surgery. During implantation of the foldable lens, one haptic of the intraocular lens fractured at the optic haptic junction. This caused lens decentration, necessitating an intraocular lens exchange ten days later. The patient was recovering well but presented again two weeks later with a drop in vision due to corneal edema. A retained haptic of the intraocular lens was seen in the inferior anterior chamber angle. After surgical removal of the retained haptic the corneal edema fully resolved. Retained intraocular lens fragments can cause corneal endothelial damage, which may be reversible with an early diagnosis and intervention.

Corneal edema after cataract surgery usually presents immediately postoperatively and often improves over a period of time in most patients. We present an unusual case where corneal edema developed one month after a complicated cataract surgery.

## Case Report

**Figure 1:**
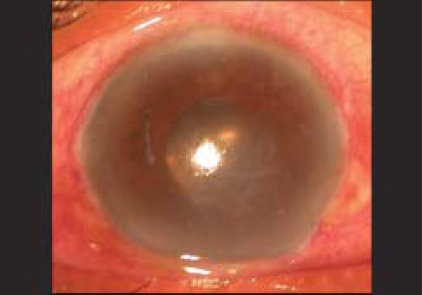
Preoperative picture showing corneal edema in the lower cornea

**Figure 2:**
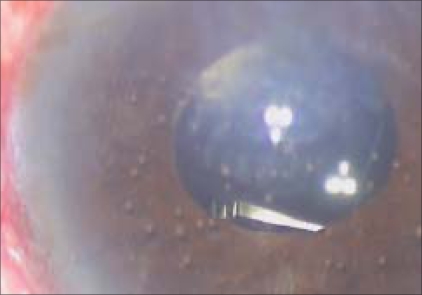
Intraoperative picture showing the intraocular lens fragment in the anterior chamber

**Figure 3:**
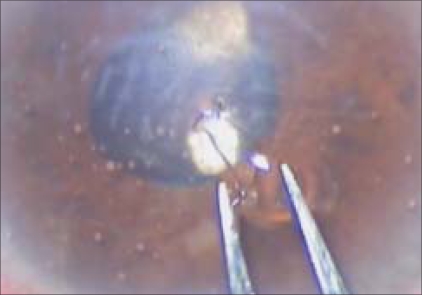
Intraoperative picture of the explanted intraocular lens fragment

**Figure 4:**
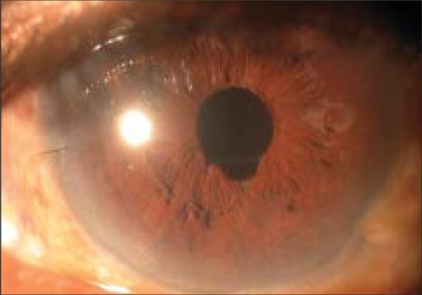
Postoperative picture at six weeks showing a clear cornea

A 65-year-old patient was referred with slowly progressing corneal edema after a complicated cataract surgery. The patient had undergone phacoemulsification with foldable intraocular lens (IOL) implantation (Rayner Centerflex 570C +21D) one month before presentation. During implantation one haptic of the IOL fractured at the optic haptic junction. Due to non-availability of a second IOL, the IOL was not exchanged. Postoperatively the patient developed IOL decentration and the IOL was exchanged on the tenth postoperative day. The IOL was explanted in a piecemeal fashion through the original incision. A new foldable IOL of the same specification was implanted using a disposable injector supplied with the lens. The patient was recovering well after the IOL exchange achieving a best corrected vision of 20/20. The patient presented two weeks later with a drop in vision. On examination, corneal edema involving the lower half of the cornea was noted by the operating surgeon and the patient was referred for cornea evaluation. On slit-lamp examination there was corneal edema [Fig. 1] involving the inferior half of the cornea, there were no keratic precipitates and no evidence of a Descemet's membrane detachment. A careful examination revealed a small piece of the IOL haptic in the inferior anterior chamber angle. On dilated examination the intraocular lens was in the bag with both haptics *in situ* and the posterior capsule was intact. Fundus examination was normal. No other lens or nuclear fragments were seen. Removal of the IOL haptic was advised and it was performed four days later. Under peribulbar block a side port incision was made and viscoelastic was used to viscoexpress [Figs. 2 and 3] the broken haptic. The viscoelastic was removed and the side port was closed by one 10–0 nylon suture. Postoperatively gatifloxacin 0.3% with dexamethasone sodium phosphate 0.1% eye drops (Gatilox-DM, Sun Pharma, India) were prescribed six times daily and hypertonic saline 5% (Hypersol - 5, Jawa Pharma, India) eye drops four times daily. Six weeks postoperatively the cornea was clear [Fig. 4] and the patient achieved best corrected visual acuity of 20/20.

## Discussion

Late onset of corneal edema after a cataract surgery has been reported to be caused by cataract nuclear fragments,[1] IOL fragments[2] and rarely by endotheliitis.[3] Damage to a foldable IOL can occur during the injection process and this may necessitate an IOL exchange. Explantation of a foldable IOL without enlarging the phacoemulsification incision usually requires cutting of the IOL to explant it. Each piece must be meticulously removed. Any missed fragment, if left inside may cause progressive corneal endothelial damage and present as corneal decompensation.

Explantation of a foldable IOL through the original incision is challenging. In a large series of 52 cases[4] the perioperative complications included zonular dehiscence and posterior capsule rupture (23.1%), total capsule-IOL extraction (7.7%), iridodialysis (5.8%), hyphema (3.8%), and retained haptics (9.6%). A meticulous technique is important to prevent inadvertent damage to intraocular structures and also to avoid leaving behind a fragment in the eye. The IOL optic can be folded inside the eye[5] with folding forceps and then explanted without enlarging the wound. This technique minimizes damage to the posterior capsule and corneal endothelium. Trisection[6] through a small clear corneal temporal incision has also been shown to be a simple and safe method of explantation. Due to the elastomeric properties of silicone, safe explantation of a silicone lens[7] in one piece, has been described by deforming the optic prior to explantation.

Corneal edema due to a retained fragment begins in the inferior cornea and slowly progresses centrally and may progress to a pseudophakic bullous keratopathy. Irreversible corneal edema has been reported as early as one month after IOL exchange.[2] It was only at the time of the corneal transplant, that the causative silicone fragment was discovered and removed from the anterior chamber. A detailed slit-lamp examination including gonioscopy is mandatory for patients who develop corneal edema with or without an inflammatory reaction in the first few weeks after an uneventful early postoperative period. Presence of a dense arcus and corneal edema may make visualization difficult. The corneal edema can be cleared temporarily with drops of Glycerol 50%. This will facilitate slit-lamp biomicroscopy and gonioscopy in an attempt to find any offending particles.

Corneal graft failure secondary to a broken posterior chamber polymethyl methacrylate IOL haptic[8] necessitating a regraft with an IOL exchange has also been reported.

Our case highlights the need for a meticulous technique of IOL explantation and a high index of suspicion for retained IOL fragment in a patient developing late corneal edema after an IOL exchange. Early diagnosis and removal of the IOL fragment prevented irreversible corneal edema in our case, as has been reported earlier.[2,8]

## References

[1] Hui JI, Fishler J, Karp CL, Shuler MF, Gedde SJ (2006). Retained nuclear fragments in the anterior chamber after phacoemulsification with an intact posterior capsule. Ophthalmology.

[2] Hoffman RS, Fine IH, Packer M (2004). Retained IOL fragment and corneal decompensation after pseudophakic IOL exchange. J Cataract Refract Surg.

[3] Olsen TW, Hardten DR, Meiusi RS, Holland EJ (1994). Linear endotheliitis. Am J Ophthalmol.

[4] Gashau AG (2006). Hydrophilic acrylic intraocular lens exchange: Five-year experience. J Cataract Refract Surg.

[5] Geggel HS (2000). Simplified technique for acrylic intraocular lens explantation. Ophthalmic Surg Lasers.

[6] Por YM, Chee SP (2007). Trisection technique: A 2-snip approach to intraocular lens explantation. J Cataract Refract Surg.

[7] Batlan SJ, Dodick JM (1996). Explantation of a foldable silicone intraocular lens. Am J Ophthalmol.

[8] Eleftheriadis H, Sahu DN, Willekens B, Vrensen GF, Liu CS (2001). Corneal decompensation and graft failure secondary to a broken posterior chamber polymethyl methacrylate intraocular lens haptic. J Cataract Refract Surg.

